# Macaques are risk-averse in a freely moving foraging task

**DOI:** 10.1038/s41598-019-51442-z

**Published:** 2019-10-21

**Authors:** Benjamin R. Eisenreich*, Benjamin Y. Hayden, Jan Zimmermann

**Affiliations:** Department of Neuroscience, Center for Magnetic Resonance Research, and Center for Neuroengineering University of Minnesota, Minneapolis, MN 55455 USA

**Keywords:** Neuroscience, Zoology

## Abstract

Rhesus macaques (*Macaca mulatta*) appear to be robustly risk-seeking in computerized gambling tasks typically used for electrophysiology. This behavior distinguishes them from many other animals, which are risk-averse, albeit measured in more naturalistic contexts. We wondered whether macaques’ risk preferences reflect their evolutionary history or derive from the less naturalistic elements of task design associated with the demands of physiological recording. We assessed macaques’ risk attitudes in a task that is somewhat more naturalistic than many that have previously been used: subjects foraged at four feeding stations in a large enclosure. Patches (i.e., stations), provided either stochastically or non-stochastically depleting rewards. Subjects’ patch residence times were longer at safe than at risky stations, indicating a preference for safe options. This preference was not attributable to a win-stay-lose-shift heuristic and reversed as the environmental richness increased. These findings highlight the lability of risk attitudes in macaques and support the hypothesis that the ecological validity of a task can influence the expression of risk preference.

## Introduction

Many animals, including humans, prefer sure things to gambles^1^. The tendency to minimize risk, i.e. unknowable and unpredictable variation, has been a topic of interest from behavioral ecology^2,3^ to economics^4,5^ and neuroscience^6–11^. Furthermore cognitive processes related to decision making in risky contexts underlies many maladaptive behaviors such as addiction and problem gambling^12,13^. Consequently, understanding risk attitudes in varying contexts provides important insight into the evolutionary origin, and thus the psychological and neural mechanisms, of addiction and maladaptive choice^14^.

Theoretical and experimental work on risk preference in non-human animals has delineated risk-aversion as a default preference for many species^1,15–19^. However risk preferences may not be as rigidly fixed as we might imagine; several factors have been demonstrated to shift risk-preference. Internal factors related to energetic states and metabolic processes are facultative on risk-preferences^17,20–23^. When faced with the possibility of starvation, many species will increase their tolerance for risk^17,21,24^. Likewise external factors related to the environmental richness, that is how much food is readily available, shift risk tolerance^16,25–27^. Risk preferences are also sensitive to the reward rate, both the timing of delivery and overall size of rewards^15,28–31^. Lastly, whether risk is explicitly cued or learned through experience impacts the expression of risk-preference^32–34^.

Rhesus macaques, the predominant model in neuroscience for understanding human decision making, are robustly risk-seeking in a variety of contexts^6,8,35–39^. Risk-seeking in macaques persists even when factors known to shift risk preferences are manipulated. For example, altering the cost of engaging in risk-seeking by increasing the inter-trial-interval reduces, but does not eliminate, macaques’ preference for risk^35^. In fact, only one study we know of has reported risk-aversion in rhesus macaques^40^.

Explanations for why macaques exhibit robust risk-seeking in experimental tasks come in two types. One type of explanation assumes that macaques’ risk attitudes are an evolved reflection of their foraging history. This view is supported by observed patterns of risk-seeking in primate species across a variety of experimental methods^41–44^. Another possibility is that macaques’ risk-seeking is a consequence of experimental tools typically used to measure their risk preferences. The manner in which macaques’ risk attitudes are measured is generally different from methods used for other species^3,45,46^ but it remains unclear how influential that difference is on general risk attitudes. The majority of data on macaque risk preference comes from studies tailored to the needs of electrophysiology, not cross-species comparison. Thus they are tested with rapid trials, often as fast as three seconds per trial, extremely small stakes, abstract stimuli, immediate rewards, overtraining, oculomotor responses, and hundreds or thousands of trials in a few hours. It may be that one of these factors, or some combination thereof, motivates risky choice. Indeed, even humans can become risk-seeking when gambling for small rewards in conditions designed to be similar to those used in non-human primate experiments^34,45^.

For foraging animals, risk manifests as an embedded component of their environment^15,28,47^. A macaque foraging for fruit may experience risk as variation in the likelihood of encountering patches of fruit-bearing trees or as variation in the quality and quantity of fruit located at an individual tree. In the former, risk may affect the decision on where to search and which tree to climb, while the latter may impact the decision for how long to reside within a particular patch or tree. Risk is also mitigated or exacerbated by the local dynamics of the foraging environment. These can include both the environmental richness and the movement costs related to the spatial position of food patches^48,49^. When food is plentiful the energetic cost of engaging in riskier foraging strategies is minimized^27^. Evolutionary pressures are believed to have shaped the cognitive architecture of foragers to navigate risk within nature and the degree to which experimental tasks match onto the natural dynamics of environment likely impacts the expression of risk^50–52^.

We hypothesized that embedding the experience of risk within a more naturalistic setting would result in macaques expressing risk preferences opposite to the trend of robust risk-seeking. We designed a naturalistic foraging task based on the patch-leaving problem from foraging theory^2,53,54^. We tested subjects (n = 3) using a single subject design within a large enclosure that allowed for free movement between four different feeding stations. Our task design incorporates risk within the stochasticity of patch harvest rates. Thus, we are able to examine the influence of risk across the use of patch types in addition to within particular patches. We found that macaques were risk-averse under these foraging conditions. We are able to abolish risk-averse preferences by increasing the overall richness of the environment in relation to the amount of variation in a risky patch. Two of the same subjects exhibited risk-seeking in a standard risk task designed for physiological recording, indicating that their risk preferences are task-specific, not individual-specific. Taken together, our results demonstrate the effect of the environmental structure on the expression of risk attitudes in rhesus macaques and highlight the importance of using naturalistic tasks for studying cognitive processes.

## Methods

### Subjects and apparatus

Three male rhesus macaques served as subjects for the experiment. Two of the subjects (C and K) had previously served as subjects on standard neuroeconomic tasks, including a set shifting task^55^, a diet selection task^56,57^, intertemporal choice tasks^58^, and a juice gambling task^10^, while the third subject (Y) was naïve to all experimental procedures. All three subjects were fed ad libitum and pair housed within a light and temperature controlled colony room. Subjects were water restricted to 25 mL/kg for initial training, and readily worked to maintain 50 mL/kg throughout experimental testing. All research and animal care was conducted in accordance with University of Minnesota Institutional Animal Care and Use Committee approval and in accord with National Institutes of Health standards for the care and use of non-human primates.

**Figure 1:**
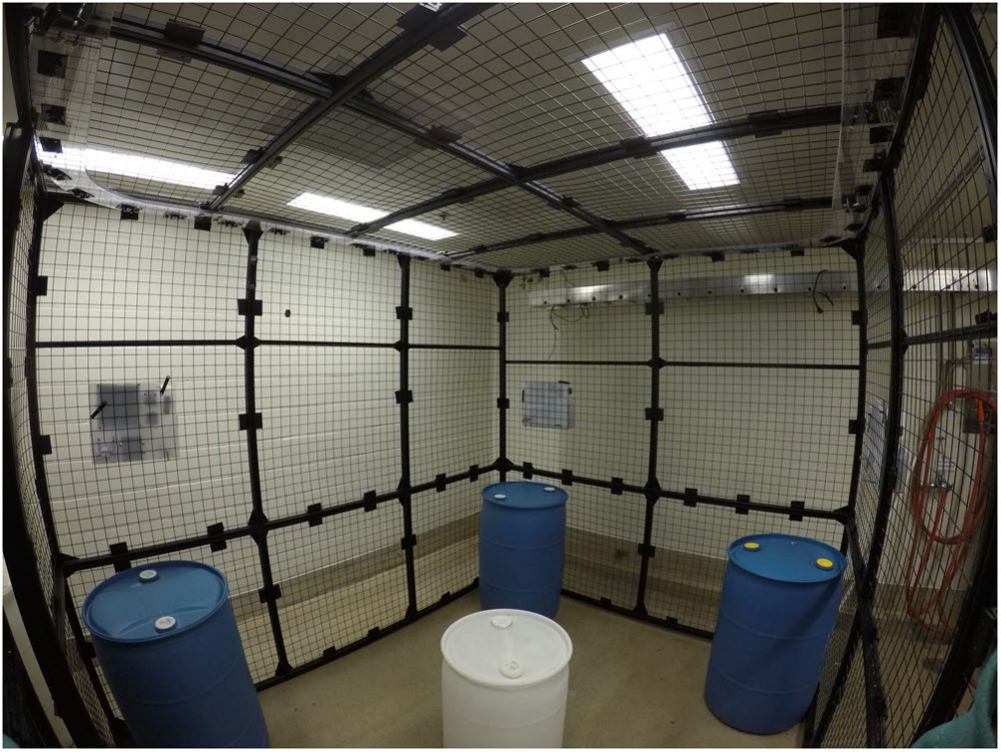
Subjects were tested within a large wire mesh enclosure. Juice feeders, attached to the walls of the cage in each corner, provided experimental stimuli and rewards. Five barrels served as perches for subjects to sit on during experimental testing.

Subjects were behaviorally tested in a large cage (~3 m × 3 m × 3 m) made from framed panels consisting of 5 cm wire mesh (Fig. 1). This allowed for free movement of the subjects within the cage in three dimensions. Five 208 L drum barrels weighted with sand were placed within the cage to serve as perches for the subjects to sit upon. Four juice feeders were placed at each of the four corners of the cage in a rotationally symmetric alignment. The juice feeders consisted of a 16 × 16 LED screen, a lever, buzzer, a solenoid (Parker Instruments), and were controlled via an Arduino Uno microcontroller. Data were collected in MatLab (Mathworks) via Bluetooth communication with each of the juice feeders.

### Training

Previous training history for two these subjects included two types of foraging tasks^57,59^, intertemporal choice tasks^34,60^, two types of gambling tasks^10,61^, attentional tasks similar to those in^62^, and two types of reward-based decision tasks^63,64^.

We first introduced subjects to the large cage and allowed them to acclimate to it. Acclimation consisted of placing subjects within the large cage for progressively longer periods of time over the course of about five weeks. To make the cage environment more positive, we provisioned the subjects with copious food rewards (chopped fruit and vegetables) placed throughout the enclosure. This process ensured that subjects were comfortable with the large cage. We then trained subjects to use the juice dispenser. All three subjects were initially trained to lever press for juice rewards in the testing enclosure. Acquisition of reliable lever pressing took about three weeks. We defined acquisition as obtaining juice rewards in excess of their daily water minimum. After completing lever training, we placed subjects onto the first experimental condition of the freely moving patch-leaving task.

### Experimental testing

Working with captive non-human primate subjects imposes unavoidable practical limitations on the number of available subjects. We therefore structured our research design and analysis around a single subjects approach^65^. Formally, we used a multiple baseline approach for collecting and analyzing behavior in the freely moving patch-leaving task^66^. We tested subjects on the first experimental condition until five days of consistent behavior were observed. This training period also served as the initial learning period for the task contingencies. The criterion of five days was chosen a priori based on previous studies using foraging tasks^59,67^. This criterion ensured that the subjects were well trained and had ample opportunity to learn the task contingencies. We defined consistent behavior as similar allocation of lever presses at a juice feeder across days. We measured behavioral consistency as the total amount of juice collected at each feeder across days within a criteria of +/− 5 mL. After observing five days of consistent behavior, we then tested subjects for an additional five days. We then implemented the second experimental condition and repeated the same observation sequence. Throughout both experimental manipulations we used the same criterion of five days of consistent behavior, as a metric for ensuring subjects understood the experimental contingencies within a condition and were at a stable state of responding. All subjects were tested in the same order experiencing the standard environmental condition first and then the rich condition. Post hoc analyses of subject behavior revealed no significant changes across the five days of experimental testing. We performed all analyses on the five days of testing after establishing consistent behavior.

### Behavioral tasks

#### Freely moving patch-leaving task

**Figure 2:**
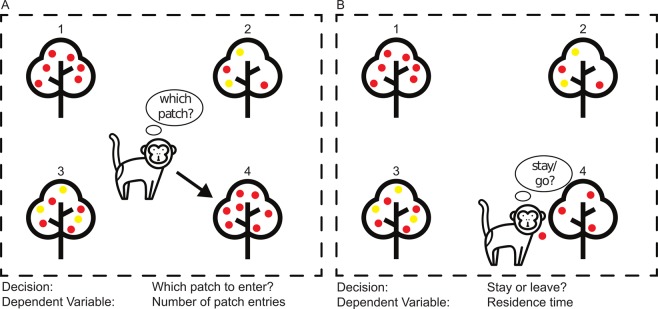
Cartoon depiction of the freely moving patch-leaving risk task design and structure. (**A**) Subjects choose between four possible patches, two safe and two risky. Risk preferences manifest in the allocation of patch entries between the two patch types. (**B**) Once in a patch subjects receive rewards according to the predefined reward schedule and must choose to either leave or remain in the patch. Risk preferences at this state are expressed as different patch residence times between risky and safe patches.

The freely moving patch-leaving task incorporates the dynamics of the natural environment by using multiple patches and a reward schedule designed to mimic the natural depletion of prey items from a patch the longer a subject forages from it^57,67^ (Fig. 2**)**. Two of the four feeders diagonally across from each other were designated as variable (risky) feeders, while the other two served as safe feeders and had no variation in reward delivery. Feeders were visually identical, although they could be readily discriminated by their position relative to landmarks outside the cage. The feeder designations remained spatially fixed for each subject across experimental days. Each feeder displayed the total amount of juice available within the patch via a blue bar (8 × 16 LEDs). With each lever press, juice would be delivered and a portion of the blue bar would disappear, explicitly indicating its depletion status. Leaving a feeder to activate any of the other three feeders would cause the previously activated feeder to immediately fully replenish, cued by a full bar being displayed. Subjects were placed within the testing enclosure and allowed to forage freely between the four feeders for two hours each day.

#### Patch reward statistics and risk

**Figure 3:**
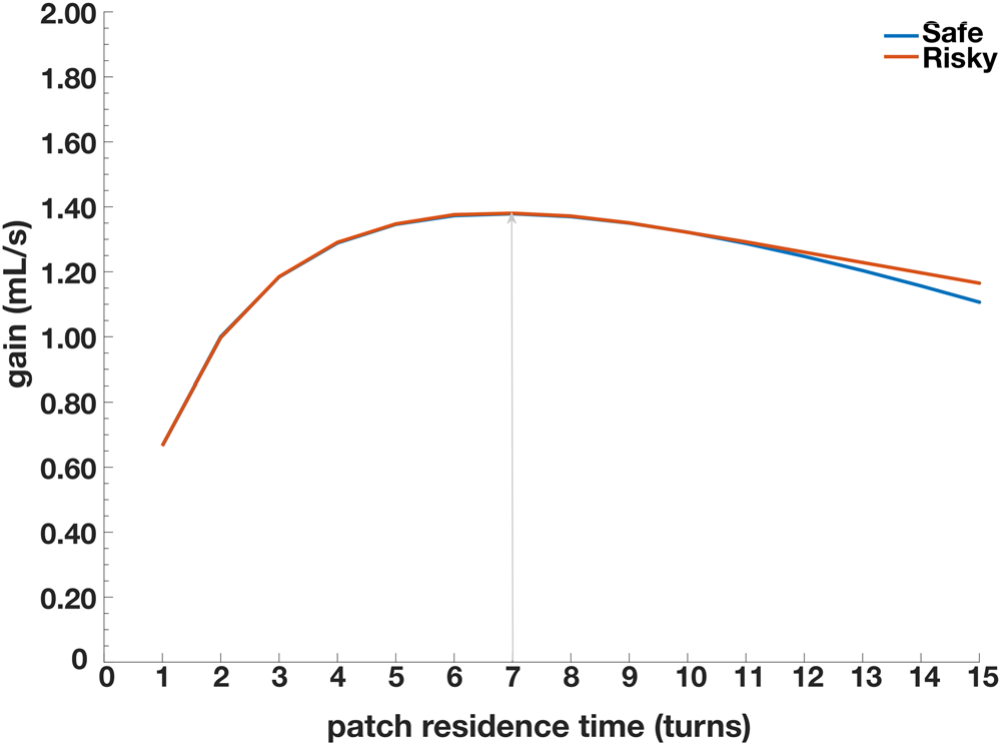
Gain function, rate as a function of residence time, for safe patches (blue line) and risky patches (red line). The black arrow denotes the abscissa point of the maximum intake rate, and thus the rate-maximizing strategy for both patch types. Due to the programed variation in reward amounts, the gain function for risky patches diverges slightly from the safe patch at long residence times. This divergence arises due to the limitation of reward amounts being bounded at 0 seconds of solenoid open time.

Each feeder was programed to deliver a base reward schedule that decreased by a specified amount. In the standard condition each feeder delivered a base reward consisting of an initial 2 mL of juice that decreased by 0.125 mL with each subsequent delivery (turn). In the rich condition, the feeders provided 4 mL of juice that decreased by 0.25 mL each turn (Table 1**)**. Risk, here defined as variation in reward amounts, was introduced by programming two of the juice feeders to randomly increase or decrease the juice delivery amount by 1 mL in addition to the base reward schedule at a probability of 0.5. Thus on any given turn, a response at the risky feeder may produce more or less juice, including non-reward delivery, than a safe feeder. Both feeder types delivered rewards following their respective schedules until reaching the base value of 0, at which point the patch is depleted and no more rewards were delivered. In practice this depletion process results in identical gain functions over the majority of patch residence times, that is the long run expectation of both feeder schedules are identical. However because the schedule had a bound at 0 mL, the tail end of the gain function for risky patches does diverge from safe patches (Fig. 3).

**Table 1:** Reward schedules for safe patches and risky patches across the two environmental manipulations, standard and rich.

		Number of Turns															
		1	2	3	4	5	6	7	8	9	10	11	12	13	14	15	16
Standard Environment	Safe	2	1.875	1.75	1.625	1.5	1.375	1.25	1.125	1	0.875	0.75	0.625	0.5	0.375	0.25	0.12
	Risky Win	3	2.875	2.75	2.625	2.5	2.375	2.25	2.125	2	1.875	1.75	1.625	1.5	1.375	1.25	1.12
	Risky Loss	1	0.875	0.75	0.625	0.5	0.375	0.25	0.125	0	0	0	0	0	0	0	0
Rich Environment	Safe	4	3.75	3.5	3.25	3	2.75	2.5	2.25	2	1.75	1.5	1.25	1	0.75	0.5	0.25
	Risky Win	5	4.75	4.5	4.25	4	3.75	3.5	3.25	3	2.75	2.5	2.25	2	1.75	1.5	1.25
	Risky Loss	3	2.75	2.5	2.25	2	1.75	1.5	1.25	1	0.75	0.5	0.25	0	0	0	0

The probability of winning or loosing at risky patches for both environments and across turns was held constant at 0.5. For all reward schedules responding after the 17^th^ turn produced no reward.

#### Definition of risk preference

Our task design defines risk preference as frequency of risky decisions made by an animal. We defined the proportion of patch entries greater than chance into risky patches as risk-seeking, and the inverse of that as risk aversion. Equal entry into both patch types was considered risk neutral. For patch residence time we defined risk-seeking as a significant tendency to stay longer in risky patches than safe ones, and risk aversion as the opposite of this trend.

#### Coefficient of variation

Rich environments in which food sources are abundant have been demonstrated to increase risk-seeking foraging strategies^27^. The coefficient of variation describes this effect as the relationship between experienced variation and the overall mean reward rate. In many species risk-seeking increases as the coefficient of variation decreases^25,26,68^. We manipulated the coefficient of variation by increasing the overall rate of reward from 2 mL with a decay of 0.125 mL/lever press to 4 mL with a decay of 0.25 mL/lever press while holding the variation constant at +/− 1 mL. Importantly this manipulation does not change the overall expectation of the reward schedules; they are still matched for both risky and safe patches.

#### Juice gambling task

**Figure 4:**
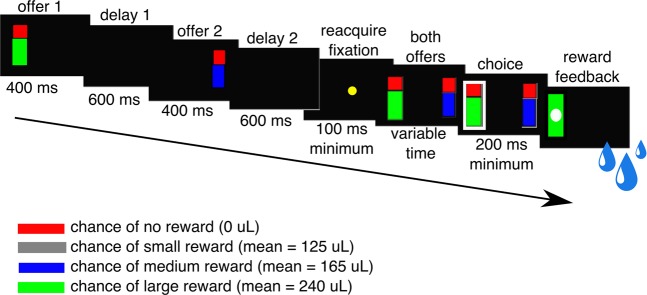
Timeline of the juice gambling task. Offers were presented asynchronously and signaled different gambles for water rewards. Offer stakes were represented by the rectangle’s color (gray, blue, green), while probability was indicated by the size of an overlapping red bar.

Data from the juice gambling task (Fig. 4), which we used as a comparison, were previously collected for electrophysiology experiments^10,69,70^ and only available for subjects C and K. In brief, the task consisted of paired choices presented rapidly (~3 sec duty cycle) while subjects sat in a specially designed chair (Christ Instruments, Hagerstown, MD). On each trial offers were presented asynchronously. The first offer appeared for 400 ms either on the left or right with equal probability. A blank period of 600 ms followed. Then the second offer appeared for 400 ms, followed by another 600 ms blank period. Following a brief central fixation period, subjects expressed their choices with saccades to the presented offers. Offers were colored bars that indicated probability and stakes. The stakes were indicated by the color of the displayed bar indicating a base reward amount (red = 0 μL, grey = 125 μL, blue = 165 μL, green = 240 μL). The probability was drawn from a uniform distribution and indicated by the height of an overlapping red bar. For example a blue bar covered halfway with a red bar represents a probability of 0.5 for receiving the reward corresponding to the color blue. Within the juice gambling task, risk is characterized as trial-to-trial variation in the probability of receiving a particular reward amount. Subjects were well trained on the task, having completed over 10,000 trials across many sessions before electrophysiological recording. For analysis we chose a random set of five days from the period of electrophysiological recording.

### Data analysis

We focused our analysis of the freely moving patch-leaving task on the five days of testing after the initial learning period in both the standard and rich condition. Drawing from our experimental design, we restricted our analyses to changes within each individual subject’s behavior. A key strength of this approach lies in our ability to rule out individual differences as an explanation for behavioral changes, as each subject serves as their own control. Furthermore, each subject serves as a replication of the previous differing only in the inter subject domain. This allows us to infer strong causal relationships between our experimental manipulations and the subsequent behavior of our subjects.

#### Freely moving patch-leaving risk task

For the freely moving patch-leaving risk task we recorded lever presses at each of the four juice feeders throughout the 2-hour testing session. We defined patch entries as a lever press at a patch different from the previously recorded lever press. We defined consecutive lever presses or turns at a juice feeder as the patch residence time. Each daily session consisted of multiple patch entries at each of the four feeders of variable patch residence times. Data from daily sessions were combined across the five days following the initial learning period for each subject within the experimental condition.

We analyzed the differences in the proportion of subjects’ patch entries between risky and safe patches across the two conditions of environmental richness using an 1-factor *ANOVA*. We investigated subjects’ risk preferences on patch residence times across the manipulation of environmental richness using a 2-factor *ANOVA* (patch type × environmental richness). We analyzed differences in patch residence times between risky and safe patches using unpaired *t-*tests. To examine if subjects used a win-stay-lose-leave strategy we examined the effect of reward outcomes one turn back and two turns back from the end of each patch residency within risky patches.

#### Risk parameter estimation

A second way to categorize risk preferences is to examine the utility function derived from the expressed choices of subjects^40,71^. To analyze differences in risk preferences between the juice gambling task and our patch leaving risk task we fit each subject’s choice preferences for offer 1 from the juice gambling task or for the decision to stay in the current risky patch to the two equations below (Eqs 1 and 2) using maximum likelihood estimation. Equations 1 and 2 produce expected utility curves whose shape is dictated by the parameter alpha. The parameter *α* functions as an index of risk preference such that *α* < 1 indicates risk-aversion, *α* > 1 indicates risk-seeking, and *α* = 1 risk-neutrality. Graphically a value of *α* = 1 will produce a straight line in which all reward amounts are equally weighted. Values of *α* < 1 produce a concave utility curve in which larger rewards undergo diminishing returns, while values of *α* > 1 produce convex utility curves in which larger rewards are given greater weight. The parameter b in both equations represents the slope of the sigmoid choice function around the point of indifference, p(choice) = 0.5. As such, b provides a measure of variation in choice.

1 $$Juice\,Gambling\,Task\,p(choice\,|offer\,1)=\frac{1}{1+(\exp ((p1\ast v{1}^{\alpha })-(p2\ast v\,{2}^{\alpha })\ast b)}$$

where:

p1 = probability of offer 1

v1 = value of offer 1 (s)

p2 = probability of offer 2

v2 = value of offer 2(s)

*α* = risk preference index

b = measure of choice stochasticity

2 $$Patch\,leaving\,Risk\,Task\,p(stay|t)=1/(1+exp(threshol{d}^{\alpha }-V{(t)}^{\alpha })\ast b)$$

where:

t = time measured in discrete lever presses

threshold = point of indifference between staying and leaving a patch

V(t) = current reward amount available given the time spent in the patch

a = risk preference index

b = measure of choice stochasticity

## Results

### Macaques spend more time in safe patches in a standard environment

**Figure 5:**
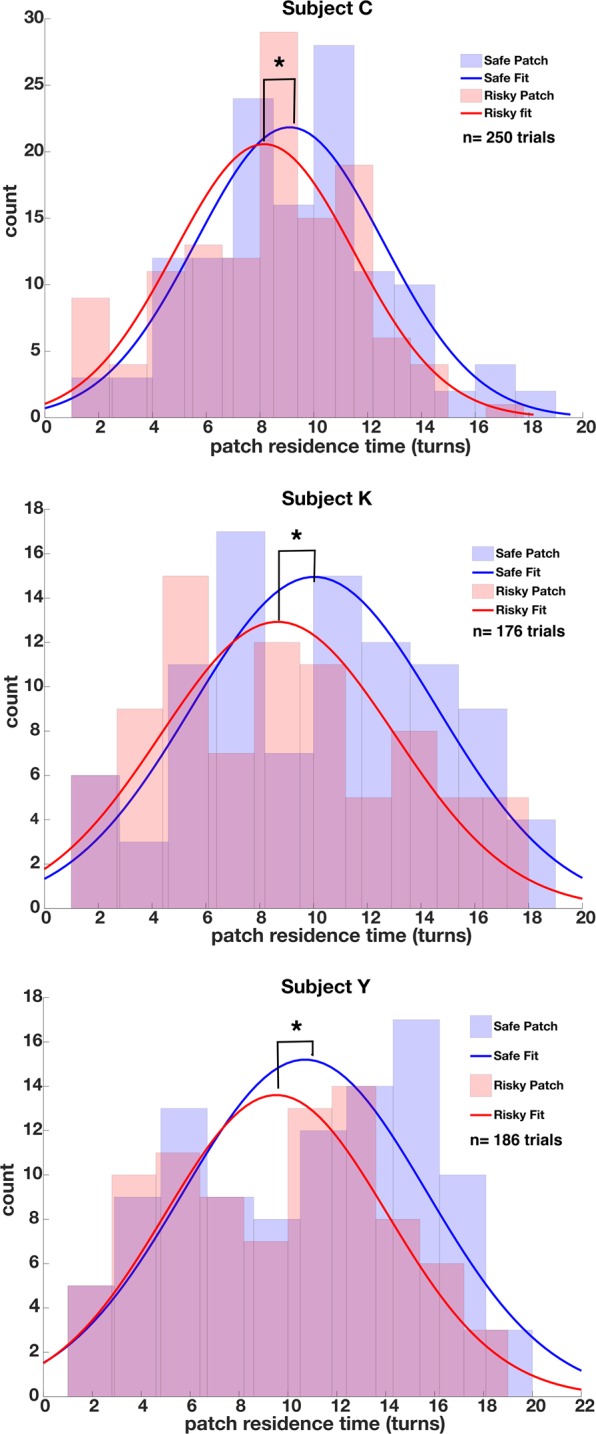
Histograms of recorded patch residence times for all subjects in safe (blue) and risky (red) patches for the standard environment condition. Residence time is indexed as the turn length or number of consecutive lever presses at a given patch before leaving (x-axis). The y-axis denotes the number of times a particular turn length occurred at the patch type. Solid lines indicate Gaussian fits to the observed leaving times. Patch residence times are significantly longer for safe than risky patches, indicating risk aversion.

We examined patch residence times in safe and risky patches defined as the number of turns spent at a feeder. Within the standard environment, all three subjects remained in the safe patches (turn means C: 9.10, K: 10.03, Y: 10.70) longer than in the risky (turn means C: 8.15, K: 8.67, Y: 9.52) ones (Fig. 5, unpaired *t-*test C: 0.9479 turns, *t*(248) = 2.198, *p* = 0.0144, d = 0.278; K: 1.35 turns, *t*(176) = 2.0289, *p* = 0.022, d = 0.304; Y: 1.17 turns, *t*(184) = 1.6842, *p* = 0.0469, d = 0.247). That is, all three subjects made more consecutive lever presses in safe patches than risky ones.

### No evidence for win-stay/lose-shift heuristic in guiding patch-leaving

It is possible that macaques’ longer residence times in safe patches are due to a data censoring effect: perhaps they leave when any individual outcome is lower than some threshold. That is, they may obey a win-stay lose-shift heuristic^72–75^. To determine if subjects used this heuristic, we examined the likelihood of leaving a risky patch given the recent history of wins and losses. None of the three subjects exhibited a significant preference of increased patch-leaving immediately after losses (one sample *t-*test C: *t*(122) = 1.1740, *p* = 0.2427, K: *t*(82) = 0.5465, *p* = 0.5862, Y: *t*(85) = 0.6448, *p* = 0.5208). Nor did we observe any effect of harvest outcomes two steps back (*ANOVA* C: *F*(3,119) = 0.83, *p* = 0.8009, K: *F*(3,79) = 0.13, *p* = 0.9413; Y: *F*(3,82) = 1.44, *p* = 0.237).

### Macaque risk preferences shift with the coefficient of variation

**Figure 6:**
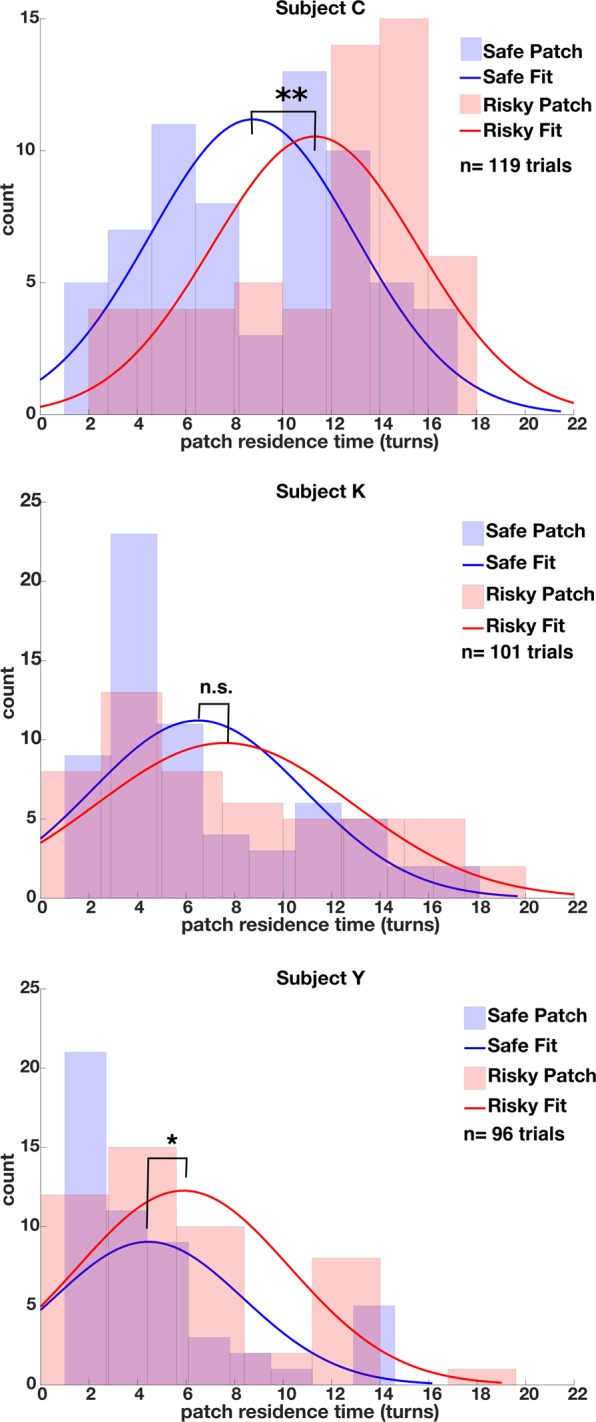
Histograms of recorded patch residence times for all subjects in the rich environment version of task. Plots follow the same conventions as Fig. 3. Subjects resided longer in risky patches than safe patches when the entire reward schedule for all feeder types was increased while maintaining the same variance as used in the standard environment.

Shifting the environmental richness serves to alter the overall mean rate of reward for the environment. When the mean rate of reward increases and variation or risk remains constant the overall coefficient of variation decreases. In all three subjects we found a significant environment by patch type interaction on their patch residence times (2-factor *ANOVA* K: *F*(1,314) = 3.1928, *p* = 0.07; C: *F*(1,376) = 18.276, *p* < 0.001; Y: *F*(1,293) = 6.7078, *p* = 0.01). All three subjects exhibited shifts away from risk-aversion to risk-neutrality/seeking as the coefficient of variation decreased (turn mean risky C: 11.32, K: 7.59, Y: 5.89, turn mean safe C:8.75, K:6.49, Y:4.44, unpaired *t-*test C: *t*(117) = 3.3303, *p* = 0.0005, d = 0.605, K: *t(*99) = 1.2077, *p* = 0.115, d = 0.226, Y: *t*(94) = 1.7483, *p* = 0.0418, d = 0.351). Thus, subjects were willing to stay longer in risky patches as the overall magnitude of reward for the environment increased relative to the variation within risky patches (Fig. 6).

### Macaques are indifferent between patch types

Foragers may choose to strategically engage with patches of a particular type as a way of avoiding variation. We found no evidence to support a preference for either patch type in any of our subjects for both standard and rich environmental conditions (1-factor *ANOVA* C: *F(1,378*) = −0.442, *p* = 0.51, K: *F*(1,316) = −0.034, *p* = 0.85, Y: *F(*1,295) = 0.01, *p* = 0.9374).

### Two of these macaques are risk-prone in computerized task

We next analyzed risky choice behavior in two subjects (C and K) in a standard (not foraging-based, not freely moving) juice gambling task^10^. Both subjects exhibited strong risk-seeking behavior. On trials with matched expected values subject C choose the risky option 67% (one sample *t*-test: t(1232) = 12.86, *p* < 0.0001) of the time, while subject K choose the risky option 66% of the time (one sample *t*-test: t(1437) = 12.55, *p* < 0.0001).

**Figure 7:**
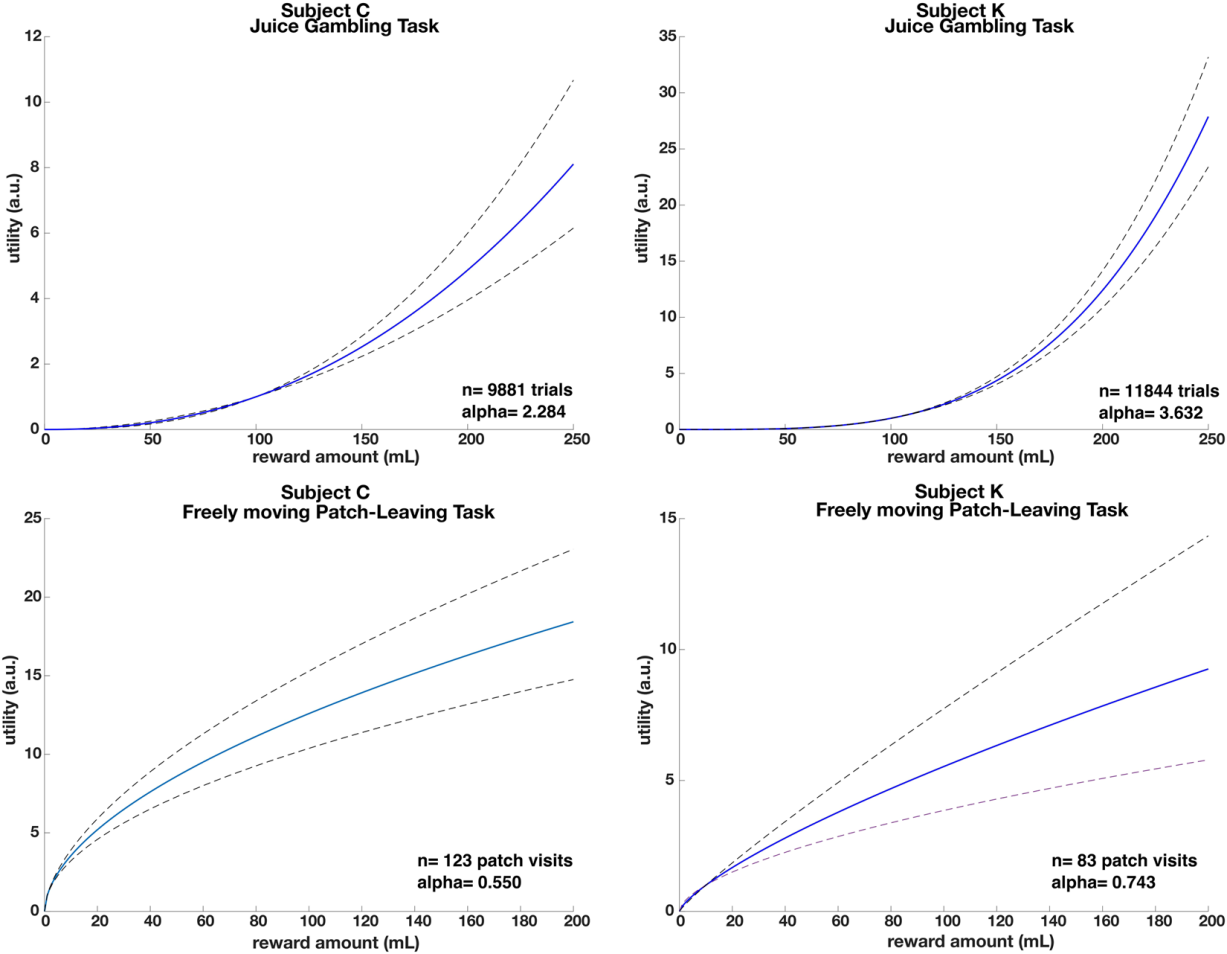
Plotted utility functions for two subjects who participated in both the feely moving patch task (lower panels) and a standard chaired economic task (upper panels). Dotted lines represent 95% CI. Two of the same macaques are risk-seeking in the standard task (convex utility curves), and risk-averse the freely moving patch task (concave utility curves).

This preference can be quantified using the shape of the utility curve. Both subjects showed convex utility curves (Fig. 7, C: alpha = 2.284, 95%CI = 2.584–1.983; K: alpha = 3.632, 95%CI = 3.822–3.441). However within the more naturalistic freely moving patch-leaving task the same subjects exhibited concave utility curves indicative of strong risk aversion (Fig. 7, C: alpha = 0.550, 95% CI = 0.5922–0.508; K: alpha = 0.743, 95% CI = 0.889–0.586).

## Discussion

Risk is ubiquitous in the natural environment and foragers must develop strategies for dealing with it^1^. There’s a general observation that animals are, for the most part, risk-averse. The earliest studies of the neurophysiology of macaque risk attitudes were problematic because they demonstrated clear risk-seeking^8,36,72,75–77^. In other words, macaques appeared to be different from other species. We hypothesize that this difference is not innate. Instead we believe it reflects the strategic adjustments macaques make when faced with the specific environment of the laboratory gambling task^18,45,46^.

To test this hypothesis, we sought to examine risk attitudes from a more complex naturalistic task. To that end, we developed a large freely moving cage apparatus with four stations, and trained our subjects to forage in from variable and stable stations, and assessed their risk attitudes.

While we would expect risk preference to manifest as a preference for using one patch type over another, we did not observe this trend. This null result can be interpreted as an expression of risk neutrality (i.e. stochastic optimality) at the level of patch choice. Foraging primates have been shown to follow simple navigation rules for moving between patches of food, and within the spatial arrangement of our task these rules would manifest as risk indifference for patch entries^78^. Future research is needed to investigate the interplay between variation in the reward rates of a patch and the spatial arrangement of patches within the environment on patch choice. We did observe that subjects remain longer in safe patches than risky ones. This increased tolerance for safe rather than risky outcomes allows us to infer that subjects value safe patches more than they value the risky ones, and demonstrates that risk attitudes are fundamentally labile. Moreover, they suggest that effort made to make the task naturalistic pays off in the form of behavior that more closely resembles that found in the wild.

Our subjects’ willingness to stay longer in safe patches as the environmental richness increases indicates that a subjective weighting of the experienced variation of rewards influences the valuation of a patch. Had subjects followed rate maximization policies under a condition of information uncertainty, we would have observed risk preferences manifest as a myopic short-term rate maximization strategies that produced a consistent censoring effect of early leaving from risky patches in both standard and rich environments^15,47,71^. One interesting question warranting further study is how the degree of information regarding the variance in reward influences the expression of risk between short-term maximization policies and the subjective weighting effects seen in conditions of pure risk.

Our results point to ostensibly minor task factors as a major component in the expression of macaque risk preferences^3,18,46^. These are the kinds of things that tend to get ignored in economic-inspired models of risky choice. Our results suggest that risk attitudes are so labile that one must carefully consider all parameters of the task design when interpreting economic preferences^79,80^. More fundamentally, these results suggest that animals may not have such a thing as a stable risk attitude. Rather, we believe that each subject has a consistent, but flexible cognitive repertoire that they use when encountering risk. In the case of rhesus macaques, their evolution and spread across diverse ecologies likely shaped their ability to adaptively shift choice strategies and preferences as environmental contingencies changed^81^. By considering how experimental tasks match onto the natural environment we can begin to fully elucidate how diverse cognitive functions such as memory, prospection, and estimation sub serve choice.

Subjects’ measured risk aversion likely does not reflect lack of training or intolerance for ambiguity. In our freely moving patch-leaving risk task subjects were well trained. Reward schedules were fixed and subjects were fully trained in the reward contingencies before testing. This represents a case of “pure risk”, in which the subject knows the reward statistics and can identify patches with variability from constant patches^71^, i.e. there is no additional ambiguity present. Furthermore, our manipulation of the coefficient of variation allows for a disassociation of reward rate strategies from subjective risk preferences in guiding patch usage, as the overall expectations of the reward schedules remains the same.

One limitation of all laboratory approaches arises out of constraints in sample size, and care should be taken with regard to any species level conclusions regarding macaque risk preference. However we are able to clearly demonstrate on a single subjects level a divergence in risk attitudes arising from the task structure. These results therefore constitute both an existence proof – that the effects we hypothesized *can* be observed in our members of the macaque species – and motivate a prediction that further studies will demonstrate a species-wide generality of these effects. In this regard, it is worth emphasizing that we did not pre-select subjects for behavior; nor did we exclude subjects for any reason.

Finally, our results call for greater effort to mimic the natural structure of the environment in order to study the evolved cognitive faculties of animals^82^. Foraging animals evolved to make decisions between foreground and background options^83,84^. Their cognitive strategies are adapted for exploiting the regularities of their natural environment, e.g. depleting patches and clumpy resource distributions^57,85–87^. It is only by carefully considering the ecological validity of our tasks that we will begin to untangle the cognitive and neural processes underlying decision making^28,60,88,89^. In this vein we join many others in arguing for greater consideration of how the environment shapes cognition and behavior^11,28,51,52,89^.

## Data Availability

All data collected and used in the analysis is available from the corresponding author upon reasonable request or can be found at www.haydenlab.com/www.zimmermannlab.com.
